# Cross-cultural validation of the “DISABKIDS” questionnaire for quality of life among Colombian children with chronic diseases^1^

**DOI:** 10.1590/1518-8345.2378.3020

**Published:** 2018-08-09

**Authors:** Nadia Carolina Reina Gamba, Miguel Richart Martinez, Julio Cabrero García

**Affiliations:** 2 PhD, Full Professor, Facultad de Salud, Universidad Manuela Beltrán, Bogotá, Colombia.; 3 PhD, Associate Professor, Departamento de Enfermería, Universidad de Alicante, Alicante, Spain.

**Keywords:** Quality of Life, Chronic Disease, Child, Adolescent, Validation Studies, Surveys and Questionnaires

## Abstract

**Objective:**

to describe the cross-cultural validation process of the “DISABKIDS” questionnaire in Colombia, for both the children and adolescents’ version and the parents’ version, an instrument intended to measure the health-related quality of life of Colombian children and adolescents with chronic diseases.

**Method:**

the cross-cultural validation process was conducted according to an international consensual systematic methodology, called standardized linguistic validation, to ensure linguistic equivalence with the original questionnaire.

**Results:**

the pretest’s cognitive interviews revealed a need to adjust the questionnaire, which consisted of asking for “health condition” rather than only asking for “condition”. Due to the cultural context, the word “condition” used in the original version, when translated to Spanish, refers to socioeconomic conditions rather than health conditions. For this reason, 11 items in the children’s version and eight items in the parents’ version were adjusted.

**Conclusions:**

the Colombian version of DISABKIDS-37 to measure health-related quality of life among children and adolescents with chronic diseases in both the children’s and parents’ versions is equivalent to the original version and is appropriate for use in Colombia. Future studies can assess the questionnaire’s psychometric properties.

## Introduction

Considering the impact of chronic diseases on children, different questionnaires are developed to measure quality of life among children and adolescents in order to better understand the impact of health problems on health-related quality of life (HRQOL), focusing on the dimensions that are most frequently affected by disease. 

Most questionnaires measuring HRQOL among children and adolescents were developed in non-Spanish-speaking countries so that the cross-cultural validation of the original versions are necessary to acquire linguistic, semantic and cultural equivalence^1^.

Standards internationally established to assess the quality of measurement instruments have determined the importance of cross-culturally validating instruments^2^ ^-^ ^3^, given the need to obtain evidence that the construct measured in the original context (in which the instrument was created) corresponds to the construct measured in the context in which it will be applied; psychometric test are expected to express such equivalence. Thus, current considerations highlight the importance of an adaptation process based on a qualitative process using various techniques, such as discussion groups and cognitive interviews in order to analyze questions such as: Is the participant thinking of what to answer? What terms does s/he use to refer to the concept? What mental effort is used to answer?^4^ ^-^ ^5^.

Consequently, despite the importance of performing a linguistic validation of health questionnaires, most papers provide brief descriptions of this process focusing only on metrical values of validity and reliability. For this reason, this paper’s objective is to describe in detail the cross-cultural validation of the “DISABKIDS” questionnaire in Colombia for the versions directed to children and adolescents and to parents, which will be used to measure HRQOL among Colombian children and adolescents with chronic diseases. 

## Methods

DISABKIDS is the first HRQOL questionnaire directed to children and adolescents with chronic diseases and their family members. It has one version for children and one for parents and was simultaneously developed in seven European countries (Germany, Austria, France, Greek, Netherlands, Sweden and the United Kingdom), with a sample of 1,153 children in the pilot test and 1,606 children in the field study, presenting robust evidence of validity^6^.

**Figure 1:**
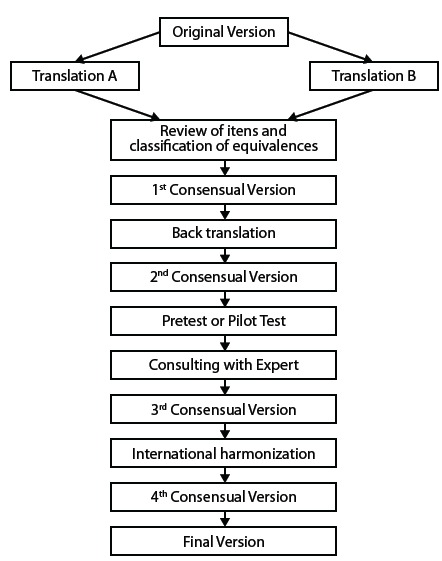
Stages of the cross-cultural validation process of the DISABKIDS - 37

Figure 1 presents the cross-cultural validation process of the DISABKIDS-37 questionnaire (children’s and parents’ versions) to measure HRQOL among Colombian children and adolescents with chronic diseases and among their parents and/or main caregivers, as indirect informants.

The study followed a consensual systematic methodology internationally called standardized linguistic validation, which is used when one desires to adapt an instrument to another language in order to ensure (semantic and conceptual) linguistic validation with the original questionnaire^1^ ^,^ ^7^. Conceptual equivalence is obtained when the answers to the same questions reflect the same concept and are meaningful in the cultures and languages involved in the process. Semantic equivalence is obtained when the selected semantic structures have the same function and the same meaning.

Cross-cultural validation Stages:

a) Direct translation. The original questionnaire was translated to Colombian Spanish (according to requirements^8^) by two independent bilingual Colombian translators who have lived in English-speaking countries and are currently living in Bogotá. They are both highly proficient in English, with knowledge of both cultures and with a professional background in the health field. The translators also assessed the degree of difficulty in translating each of the items on a scale from 0 to 10 (where 0 = no difficulty and 10 = maximum difficulty). Afterwards, the translators and the research group (researcher and dissertation advisor and co-advisors), for which discrepancies between the translated and original versions were resolved, classifying the items according to a degree of equivalence as follows: A = equivalent to the original version and there is no need to change the item; B1 = syntactic and/or semantic changes are necessary to achieve equivalence; B2 = changes concerning cultural aspects are necessary to obtain equivalence; and C = not equivalent, item is not appropriate for the Colombian context^9^. Thus, the first consensual version was agreed upon.
b) Back translation. A bilingual translator, whose mother language is English, performed the back translation. She has dual nationality (American and Swiss) and experience in Spanish-speaking countries, including Colombia (Bogotá), but is currently living in an English-speaking country. She is highly proficient in Spanish, with knowledge of both cultures and a professional background in the health field. This translator also assessed the level of difficulty to back-translate each of the items on a scale from 0 to 10 and, finally, a consensus was reached and the items were classified according to levels of equivalence, from which resulted the second consensual version.
c) Pre-test or Pilot test. Thirty-two face-to-face cognitive interviews were held by the researcher using paraphrase techniques and verbal probing method^10^, in order to achieve cognitive and cultural equivalence. The pilot test was conducted with a group of children and adolescents with chronic diseases and their mothers, fathers and/or main caregivers, in order to determine level of comprehension, clarity, and accuracy of the questionnaire items and also in regard to the answer options, determining its applicability in the cultural context. The participants consented to audio recording. The Dragon Naturally Speaking software, version 12.0, was used and the interviews were coded with a number and letter and later transcribed. A convenience sample was collected in the city of Bogotá, Colombia and 17 cognitive interviews were conducted with children and adolescents with chronic diseases, along with 15 mothers, fathers and/or main caregivers (two mothers did not consent to the audio recording but allowed their children to participate). The interviews took place in the pediatric hospitalization wards of two health facilities and a few interviews were held in the participants’ homes. Children, of both sexes, from all economic classes were aged between 8 and 18 years old and classified into three age groups. A greater number of interviews was required among the youngest ones (8-10 years old) because they generally presented greater comprehension and reading difficulties. The cognitive interviews were then analyzed and discussed, while an expert in the field of quality of life among children and adolescents and chronic diseases was consulted to resolve problem items. Thus, adjustments were implemented in the questionnaire and a third consensual version was obtained.
d) International consensus. Discussions, analyses and adjustments were necessary to obtain consensus between the original questionnaire’s authors (DISABKIDS group), led by their coordinator in Germany, and the research group. The questionnaire’s authors suggested the Brazilian version be considered, since the observations made by the children were taken into account, especially those provided by the 8-10 year-old group. Thus, the fourth consensual version was obtained and, after implementing relevant changes, the questionnaire’s final version was achieved.

The European group DISABKIDS authorized the use of DISABKIDS-37 and its adaptation, as well as its consensual final version. The study project was approved by the Institutional Review Boards at the two health facilities where the study was conducted (Numbers GCCI-033-14 and CEIFUS 1692-13 respectively) and all the participants signed free and informed consent forms.

## Results

The direct translation of the children’s and parents’ versions scored between 0 and 4 (only one item scored 4 points) in terms of difficulty of items while the back translation scored between 0 and 5.

**Figure 2:**
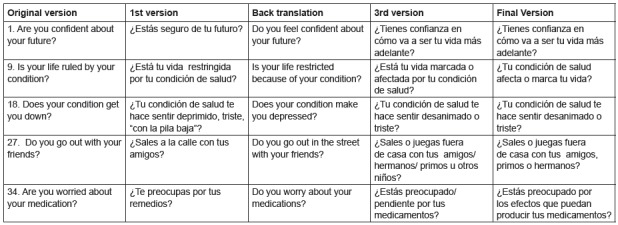
How items in the consensual versions changed.

The first and second consensual versions had 37 items. Thirty-two items in the children’s version were classified as A (semantic and conceptual equivalent to the original version; no changes required). Three items were classified as B1 (changes were necessary to obtain semantic equivalence), for example: item 9 was originally translated: “¿*Está su vida restringida por tu condición de salud?* [Is your life restricted by your health condition?], which, in the consensual version, was changed to: ¿*Está tu vida marcada/regida por tu condición?* [Is your life marked/governed by your condition?]; item 37 was translated: *“*¿*Tomar su medicación interrumpe su vida diaria?”* [Does taking medication interrupt your daily life?], but in the consensual version it was changed to: *“*¿*Tomar tus medicamentos altera/cambia tu vida?”* [Does taking medication alter/change your life?]. Two items were classified as B2 (changes concerning cultural aspects are necessary to obtain equivalence): item 18, which was originally translated: “¿Su *condición lo derrumba, deprime?*” [Does your condition knock you down/depress you?], in the consensual version was changed to “¿*Tu condición te hace sentir deprimido, triste, “con la pila baja”?* [Does your condition depress you, make you sad, leave you with low energy?”]; and item 27, which was originally translated: “¿Sales *fuera con tus amigos?*” [Do you go out with your friends?], was translated in the consensual version as: *“*¿Sales a la *calle con tus amigos?”* [Do you go out in the street with your friends?]. No item was classified as C (not equivalent). In the parents’ version, 36 items were classified as A (semantically and conceptually equivalent to original version, no need to change the item) and one item was classified as B2 (changes are needed to obtain cultural equivalence). The results concerning the consensual versions are presented in Figure 2.

**Figure 3:**
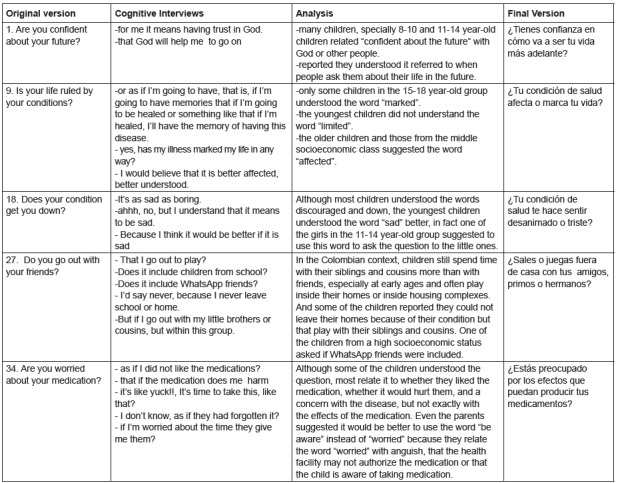
Results of the cognitive interviews held with children and adolescents during the pretest of the Colombian version of the DISABKIDS-37

Thirty-two cognitive interviews were held in the pre-test or pilot test - 17 interviews were individually conducted with children and adolescents with chronic diseases and 15 were individually conducted with their mothers, fathers and/or main caregivers (two did not consent to audio recording) in one hospital and one prepaid medicine plan in the city of Bogotá, Colombia. Two interviews held with individuals belonging to an upper economic class were conducted at their homes. 

Children were aged between 8 and 18 years old and were distributed into three age groups: 8-10 year-old group, n= 8; 11-14 year-old group, n=5; and 15-18 year-old group, n=4; girls, n=8, and boys, n=9, from all economic classes (low socioeconomic class n=9, medium socioeconomic class: n=6, upper socioeconomic class, n=2) originated from different areas of the country and presented different types of chronic disease (asthma, epilepsy, cancer, auto-immune diseases, kidney diseases). The 8-10 year-old children, of both sexes, took longer than the remaining groups to complete the questionnaire, especially those from the lowest socioeconomic class. This situation took place among children with epilepsy, while some presented mild cognitive deficit. Some of the children and adolescents and, to a lesser degree, their mothers, were somewhat nervous and shy at the beginning of the interviews but soon calmed down, so that the paraphrase techniques and verbal exploration proved to be effective. The main caregivers were mainly the mothers, followed by the fathers, grandmothers and aunties, who showed great interest in the activity. The researcher conducted the cognitive interviews over a period of two months.

In general, the results of the pretest’s cognitive interviews showed that both children and parents manifested positive impressions of the questionnaire, considering its simple, understandable language and ease of completion. A general adjustment was required: instead of asking about one’s “condition”, the term “health condition” was added. Even though the children preferred to be asked directly using the term “disease”, the term “condition” remained as determined by the authors of the original questionnaire (they preferred a neutral term instead of a stigmatizing one) so that “health condition” was chosen due to the cultural context in which the word “condition” is mainly related to socioeconomic conditions rather than health. 

As shown in Figure 2, the cognitive interviews led to the adjustment of 11 items in the children’s version and eight in the parents’ version, for instance, item 1: Are you confident about your future? The children related “*confiar en el futuro*” [trust in the future] to “*confiar en Dios*” [trust in God]. For this reason, this item remained in the final version: ¿*Tienes confianza en cómo va a ser tu vida más adelante*? [Are you confident in how your life will be in the future?]. In item 18: Does your condition get you down*?*, even though most children understood the words “discouraged” and “down”, the youngest children became confused and choose to replace the term with “sad”. For this reason, this item was translated in the final version as: ¿*Tu condición de salud te hace sentir desanimado (triste)*? [Does your health condition make you feel discouraged (sad)?]. In item 27: Do you go out with your friends?, the children asked whether it referred to playing or if it also included schoolmates or siblings and cousins, and children from higher socioeconomic classes also included WhatsApp friends, so that the final version remained: “¿*Sales o juegas fuera de casa con tus amigos (también primos/ hermanos)*?” [Do you go out and play outside home with your friends (also cousins/siblings)?]. In item 34: Are you worried about your medication?, the children related to the taste of medications or remembering to take medications, a situation that was also mentioned by the questionnaire’s authors so that, to specify that this question referred to the effects of the medications, in the final version it remained: “¿*Estás preocupado por los efectos que puedan producir tus medicamentos*?” [Are you worried with the effects your medications can produce?] and item 37: Does your child feel that taking medication disrupts his/her everyday life? The children related it with dependency or the annoyance of taking medications, so that in the final version this item remained: *“*¿*Tomar tus medicamentos altera (desmejora) tu vida diaria?*” [Does your medications alter (worsen) your daily life?].

Children older than eight years old paid attention to the questionnaire and the youngest group was the most spontaneous and participative, while the three age groups provided suggestions and observations and all children followed the instructions and answered all the questions; the children found no difficulties understanding reverse items presented on the Likert scale. Nonetheless, even though recommendations are that children be individually interviewed, most were hospitalized so that almost all mothers, fathers or main caregivers were present at the time of the interviews.

Additionally, even though the questionnaire was designed to be self-administered, special cases should be taken into account: a child may present a slight degree of cognitive impairment, given a lesion in one of the eyes (caused by the disease) or experience fatigue following chemotherapy, or may lack of glasses for instance. In cases like these, the interviewer may be required to administer the questionnaire. The same was the case for some parents or main caregivers with impaired sight or without glasses at the time. 

## Discussion

The Colombian versions of DISABKIDS-37 for children and parents, intended to measure HRQOL among children and adolescents aged between 8 and 18 years old with chronic diseases, present appropriate conceptual, semantic and cultural equivalence with the original European version in English. This study is the first to address the valuation of self-perceived health among children and adolescents with chronic diseases in the Colombian context. 

The rigorous, organized and sequential use of the international consensual methodology enabled obtaining the cross-cultural validation of the DISABKIDS-37 questionnaire for the Colombian population, the process of which included children, adolescents and their respective parents or main caregivers from some regions of Colombia. Requirements concerning the inclusion of children and adolescents based on age groups, both sexes, from all socioeconomic statuses were met in selecting the sample for the pretest. A qualitative method of cognitive interviews was also used, which was effective for assessing acceptability, comprehension, and the possibility of using better phrases to improve the understanding of items on the part of children, similar to the original study^11^. The results obtained in the pretest led to the adjustment of 11 items of the Colombian children’s version, similar to the Brazilian version, which adjusted 14 of the 37 items contained in the DISABKIDS-37. Difficulties were found in the Brazilian version translating the verbs *annoy, disrupt, make angry* and *bother* ^12^, while difficulties in the Colombian version involved *life ruled, free to lead the life, get you down, having to get help* and also *annoy* and *disrupt*. Additionally, some degree of difficulty was found in the options provided by the Likert scale in both the translation and back translation, considering that both the original version and the Brazilian version contain *quite often* and *very often*, which are almost synonymous in Colombian Spanish, thus *quite often* was translated as *“algunas veces*” [sometimes], which is an intermediate option.

The Swiss version, which was adapted among children with cancer, deleted item 17: “Do you have fears about the future because of your condition? due to the negative response of parents who were concerned that this question would make their children doubt their future, negatively affecting them^13^, a situation that was not verified to occur in the Colombian version, though it also included children with cancer and their parents. The Danish version, for instance, also addressed children with diabetes and found item 6 to be problematic: “Are you able to do things without your parents?”, a situation that was not verified in the Colombian version^14^.

The advantage of cross-culturally validating questionnaires is that it is a more economical process (in terms of time and costs) than developing a new questionnaire, in addition to facilitating comparison between different populations^15^ ^-^ ^16^. DISABKIDS has been adapted in seven European countries and in Brazil, where it has shown discriminatory capacity in pathologies such as cerebral palsy^17^, diabetes^18^ and asthma^19^ in terms of age, sex, and clinical severity, which enable making comparisons. In fact, it is currently of international interest to assess changes in HRQOL using longitudinal studies among children. A Spanish study^20^ reports that perception of quality of life verified in a healthy population of children and adolescents worsened in eight out of 10 dimensions after three years of observation, showing that girls were more impacted than boys, mainly due to puberty. Therefore, the availability of questionnaires measuring HRQOL among Spanish-speaking children is relevant. 

Cognitive interviews used in the semantic validation process show relevant information in regard to clarity, accuracy, and especially the comprehension of words and items (among children, adolescents and parents), showing results similar to those reported by other studies, such as the one conducted with children, adolescents and their parents in Portugal^21^, where item 12: Does your condition bother you when you play or do other things*?*, was considered by some Portuguese teachers (who were included in the semantic validation process) as having some degree of difficulty for younger children. The Brazilian study, similar to the Colombian study, adjusted the redaction of items 9: Is your life ruled by your conditions? and 18: Does your condition get you down? in order to obtain semantic equivalence. The Brazilian study also used the same method of cognitive interviews but added two forms of written assessment to verify general and specific impressions of the DISABKIDS-37, instead of using audio recording (which was used in this study) and also reported positive impressions and interest in the questionnaire and comprehension problems for only a few items. Therefore, the self-report version of this questionnaire is viable, even for use among the youngest children; however, special situations such as respondents with impaired sight, with no glasses available, among other possibilities, should be considered.

Nursing professionals agree that the adaptation of questionnaires is important to identifying the health-related quality of life of Colombian children and adolescents with chronic diseases in order to establish the impact of these diseases on children and adolescents and to assess the effect of nursing and health interventions and to implement measures to improve the quality of life of children and adolescents with chronic diseases. Finally, it is worthy mentioning that a limitation of this study is the long response time required to obtain authorizations from the health facilities for the fieldwork.

## Conclusions

The Colombian version of DISABKIDS-37 to measure the health-related quality of life of children and adolescents with chronic disease, whether in its version for children or for parents, is equivalent to the original version and is appropriate for use in Colombia. The following stage of this study will include assessments of its psychometric properties to determine internal and external validities.
