# Erratum

**DOI:** 10.1590/1518-8345.0000.3081

**Published:** 2018-12-03

Regarding the article “Cross-cultural validation of the ‘DISABKIDS’ questionnaire for quality of life among Colombian children with chronic diseases”, with DOI number: 1518-8345.2378.3020, published in Rev. Latino-Am. Enfermagem, 2018;26:e3020, page 1:

Where was written:

“Nadia Carolina Reina Gamba^2^

Miguel Richart Martinez^3^

Julio Cabrero García^3^”

Now Read:

“Nadia Carolina Reina-Gamba^2^

Miguel Richart-Martinez^3^

Julio Cabrero-García^3^”

